# Marfan syndrome: whole-exome sequencing reveals *de novo* mutations, second gene and genotype–phenotype correlations in the Chinese population

**DOI:** 10.1042/BSR20203356

**Published:** 2020-12-04

**Authors:** Yuduo Wu, Hairui Sun, Jianbin Wang, Xin Wang, Ming Gong, Lu Han, Yihua He, Hongjia Zhang

**Affiliations:** 1 Department of Cardiac Surgery, Beijing Anzhen Hospital, Capital Medical University, Beijing, China; 2 Key Laboratory of Medical Engineering for Cardiovascular Disease, Ministry of Education, Beijing, China; 3 Beijing Key Laboratory of Maternal-Fetal Medicine and Fetal Heart Disease, Beijing Anzhen Hospital, Capital Medical University, Beijing, China; 4 School of Biological Science and Medical Engineering, Beihang University, Beijing, China; 5 School of Life Science, Tsinghua University, Beijing, China; 6 Ultrasound Department of Beijing Anzhen Hospital, Beijing, China; 7 Department of Cardiac Surgery, Beijing Chaoyang Hospital, Capital Medical University, Beijing, China

**Keywords:** de novo mutations, genotype-phenotype correlations, Marfan syndrome, two mutation, Whole-exome sequencing

## Abstract

Marfan syndrome (MFS) is a dominant monogenic disease caused by mutations in fibrillin 1 (*FBN1*). Cardiovascular complications are the leading causes of mortality among MFS. In the present study, a whole-exome sequencing of MFS in the Chinese population was conducted to investigate the correlation between *FBNI* gene mutation and MFS. Forty-four low-frequency harmful loci were identified for the *FBN1* gene in HGMD database. In addition, 38 loci were identified in the same database that have not been related to MFS before. A strict filtering and screening protocol revealed two patients of the studied group have double mutations in the *FBN1* gene. The two patients harboring the double mutations expressed a prominent, highly pathological phenotype in the affected family. In addition to the *FBN1* gene, we also found that 27 patients had mutations in the *PKD1* gene, however these patients did not have kidney disease, and 16 of the 27 patients expressed aortic related complications. Genotype-phenotype analysis showed that patients with aortic complications are older in the family, aged between 20 and 40 years.

## Introduction

Marfan’s syndrome (MFS) is one of the most common autosomal dominant connective tissue diseases, with an incidence of 2–3/10000, caused by mutations in the fibrillin-1 gene (*FBN1*) [1]. Since 1991, when the mutation of *FBN1* was identified as the pathogenic factor of MFS [2], about 2900 variant sites have been identified in the *FBN1* gene. More than 90% of patients diagnosed with MFS revealed mutations in the *FBN1* gene [3,4]. Clinically MFS patients with *FBN1* mutation(s), express a range of phenotypes from mild to severe disease. Thus, single *FBN1* mutation cannot fully explain the heterogeneity of clinical phenotypes in MFS patients, who can express different pathologies in the eyes, skin, musculoskeletal, cardiovascular, and pulmonary systems. Despite other complications, cardiovascular pathologies remain the major risk factor for death in MFS patients [4].

The differences in the clinical phenotypes of patients with MFS exist even within the same family harboring the same genetic mutations. Moreover, the onset of the disease at different ages may further lead to different clinical phenotypes (REF). So far, no strong genotype–phenotype correlation between *FBN1* variants and MFS has been reported except for neonatal forms of MFS associated with missense variants in exons 25-33 (exons 24-32 according to next-generation sequencing technology) [5,6]. In 2017, the study of Linnea and Franken et al. indicated that patients with severe phenotypes die earlier, and the mutation usually occurs in exons 24-32; It was also found that patients with haploinsufficiency-*FBN1* mutation and *FBN1* truncation mutations had a high risk of developing an acute aortic event or requiring surgical intervention [7]. In 2019, our center conducted a study on the relationship between genotypes and phenotypes in MFS patients with aortic dissection and revealed a significantly higher frequency of frameshift and nonsense mutations exist in these patients, whereas missense mutations were at higher frequency in patients with aortic aneurysm [8].

However, the previous studies and findings thus far don't account to some clinical phenotypic differences expressed in MFS patients. Therefore, we investigated whether there are *de novo* gene sites or additional genes contributing to the phenotypic differences among MFS patients or whether there are two mutations in MFS patients.

## Materials and methods

### Sample collection and selection

The cohort consisted of 131 MFS patients, including 82 probands and their family members. Among them, there were 69 males and 62 females. The average age of the patients at the time of enrollment was 25.00 ± 14.54 (range 0.4–64), and the average age of cardiovascular involvement was 22.64 ± 14.67 (range 0–64). The diagnosis of MFS patients was performed in accordance with the 2010 revised Ghent criteria [9]. Demographic and clinical data of patients were collected from medical records of the Ultrasound Department and Department of Cardiac Surgery at Beijing Anzhen Hospital, Capital Medical University, Beijing, China. Informed consent for DNA analysis was obtained from patients in line with local institutional review board requirements at the time of collection.

### Sequencing and data analysis

#### DNA extract and sequencing

Genomic DNA extracted from peripheral blood of each patient was fragmented to an average size of 180–280 bp and subjected to DNA library creation using established Illumina paired-end protocols. The Agilent SureSelect Human All ExonV6 Kit (Agilent Technologies, Santa Clara, CA, U.S.A.) was used for exome capture according to the manufacturer’s instructions. The Illumina Novaseq 6000 platform (Illumina Inc., San Diego, CA, U.S.A.) was utilized for genomic DNA sequencing in Novogene Bioinformatics Technology Co., Ltd (Beijing, China) to generate 150-bp paired-end reads with a minimum coverage of 10× for ∼99% of the genome (mean coverage of 100×).

#### Sanger sequencing

After targeted NGS sequencing, the mutation obtained was further verified in the proband and remaining affected family members by Sanger sequencing. PCR amplification was performed using the forward primer (caactcctgtgagctgttgc) and reverse primer (acgttgtccacagtgagtcc). The obtained sequence was compared with the FBN1 reference gene (NM_000138.4) to identify mutations.

#### Data analysis

After sequencing, basecall files conversion and demultiplexing were performed with bcl2fastq software (Illumina). The resulting FASTQ data were submitted to in-house quality control software for removing low quality reads, and then were aligned to the reference human genome (hs37d5) using the Burrows-Wheeler Aligner (bwa) [9], and duplicate reads were marked using sambamba tools [10]. SNP/INDEL calling: Single nucleotide variants (SNVs) and indels were called with SAMtools to generate gVCF [11]. The raw calls of SNVs and INDELs were further filtered with the following inclusion thresholds: (1) read depth > 4; (2) Root-Mean-Square mapping quality of covering reads > 30; (3) the variant quality score > 20.

### Annotation

Annotation was performed using ANNOVAR (2017June8) [12]. Annotations included minor allele frequencies from public control data sets as well as deleteriousness and conservation scores enabling further filtering and assessment of the likely pathogenicity of variants.

### Rare variants filtering

Firstly, Filtering of rare variants was performed as follows: (1) variants with a MAF less than 0.01 in 1000 genomic data (1000g_all) [13], esp6500siv2_all [14], gnomAD data (gnomAD_ALL and gnomAD_EAS) [15] and in house Novo-Zhonghua exome database from Novogene. (2) Only SNVs occurring in exons or splice sites (splicing junction 10 bp) were further analyzed since we are interested in amino acid changes. (3) Then synonymous SNVs that were not relevant to the amino acid alternation predicted by dbscSNV were discarded; The small fragment non-frameshift (<10 bp) indel in the repeat region defined by RepeatMasker were also discarded. (4) Variations were screened according to scores of SIFT [16], Polyphen [17], MutationTaster [18] and CADD [19] software. The potentially deleterious variations were reserved if the score of more than half of these four softwares support pathogenicity of variations [20]. Sites (>2 bp) that did not affect alternative splicing were removed. Secondly, the newly discovered variants with MAF less than 0.001/0.0001 in gnomAD data (gnomAD_ALL and gnomAD_EAS) and in-house Novo-Zhonghua exome database from Novogene.

### Classification of alterations

In order to better predict the pathogenicity of the variants, this part refers to the previous research classification of our center [21]. The American College of Medical Genetics and Genomics (ACMG) variant classification recommendations were utilized for all reported variants (Richards et al., 2015). Notably, the following two conditions of missense variants were considered strong evidences of pathogenicity: “Well-established *in vitro* or *in vivo* functional studies supportive of a deleterious effect on the gene or gene product”. (1) A missense variant that created or destroyed a cysteine residue. 2) A missense variant that affected conserved residues in the EGF-like domain consensus sequence (D/N) X (D/N) (E/Q) Xm (D/N) Xn (Y/F) (m and n represent variable numbers of residues). Positive result: pathogenic or likely pathogenic variant(s) in a known disease gene associated with the reported phenotype.

#### Possible diagnosis

Variant(s) in a known disease gene possibly associated with the reported phenotype. This category includes novel variants, including missense variants or in-frame insertions/deletions in disease genes that overlap with the provided phenotype for the patients, and a single rare or highly suspicious novel variant of uncertain significance (VUS) known to be *in trans* with a pathogenic/likely pathogenic variant in a gene that explains the reported phenotype.

#### Candidate gene

Variant(s) predicted to be deleterious in a gene that has not been previously implicated in MFS or for which the published data supporting an association may not yet be definitive were considered potential novel candidates. Supporting data were based on model organism data, copy number variant data, tolerance of the gene to sequence variation, data regarding tissue or developmental timing of expression, or knowledge of the gene function and pathway analysis.

#### Uncertain result

VUS in a known disease gene and a patient phenotype consistent with the reported disease spectrum (e.g. uncertainty is limited to the pathogenicity of the variant due to a lack of parent samples to assess for *de novo* occurrence and determining the phase of variants in recessive disorders). This category also includes recessive conditions that overlap with the phenotype provided for the fetus in which only a single pathogenic/likely pathogenic variant is identified.

#### Negative result

No variants in genes associated with the reported phenotype identified.

### Analysis of genotype–phenotype correlations

In order to avoid bias for a certain locus due to the family relationship, we only extracted the proband samples from the families of enrolled MFS patients and analyzed the relationship between the genotype and the phenotype. Since some families have no proband, if there is only one member of the family, this member will be selected as the proband; if there is more than one member, a patient will be randomly selected as the proband. Finally, a set of 82 samples wereobtained. Requirement (1) frequency: reserve variants with MAF less than 0.0001 in gnomAD data (gnomAD_ALL and gnomAD_EAS) and in house Novo-Zhonghua exome database from Novogene; (2) reserve variation with functional loss (classical splicing site + - 1 / 2, code shifting, nonsense). At least two software in Sift, polyphe2, and mutationastar predict harmful missense variation; classic splicing site + - 3 to 10 mutations, retain two software predicted mutations affecting splicing [22]. At the same time, the relationship between the shared genes and disease phenotype was analyzed for some genes with a large number of shared samples.

### Statistical analysis

The quantitative data and variables are respectively presented as the mean ± standard deviation (SD) and frequencies or percentages. Quantitative variables were compared using Student’s *t* test. A value of *P*<0.05 was considered significant. Statistical analysis was performed by SPSS 22.0.

## Results

### *De novo* mutation loci in *FBN1* gene

All site information of WES in 131 MFS patients is shown in Table 1 and details of sequencing quality are shown in Table 1. Furthermore, 82 low-frequency deleterious loci were found in the *FBN1* gene (recorded in OMIM database and MFS related pathogenic genes, Table 2). Of the 82 loci, 44 loci have been reported (Supplemental Excel S1), and 38 loci have never been reported before to cause MFS according to the HGMD database (Supplementary Excel S2). In order to further improve the quality of the sequencing data, we set a higher data filtering level for the newly discovered 82 *FBN1* loci in gnomAD and Asia, and Nuohe'slocal database. The more stringent sequencing criteria yielded fewer new possibly pathogenic mutations (Table 3).

**Table 1:** Variants identified by WES in 131 samples

	SNPs	INDELs
Total	573805	98055
Frequency	209343	45239
Function	63465	4888
Exonic function	42213	3973
Deleterious	28197	3482

Sites (>2 bp) that did not affect alternative splicing were removed.

**Table 2:** Double mutation sites in the *FBN1* of MFS patients

Patients	ACMG classification	Priority	POS	Genomic	Esp 6500siv2	GnomAD	Func	ExonicFunc	AAChange (NM_000138)
**P_7**	VUS	H	48725156	-	-	-	exonic	missense SNV	exon55:c.C6646T:p.L2216F
**P_7**	LikelyPathogenic	H	48780367	-	-	-	exonic	frameshift deletion	exon27:c.3279delT:p.F1093fs
**P_33**	VUS	H	48719977	-	-	-	splicing	splicing	exon58:c.6998-7C>T
**P_33**	Pathogenic	H	48757762	-	-	-	splicing	splicing	exon40:c.4942+3-4A>G
**P_41**	VUS	H	48936888	-	-	-	exonic	missense SNV	exon2:c.G79A:p.A27T
**P_41**	Pathogenic	H	48738902	-	-	-	splicing	splicing	exon47:c.5788+1-2G>A
**P_76**	VUS	H	48780631	-	-	-	exonic	missense SNV	exon26:c.A3142G:p.I1048V
**P_76**	VUS	H	48802333	-	-	-	exonic	missense SNV	exon14:c.G1622A:p.C541Y
**P_101**	VUS	H	48780631	-	-	-	exonic	missense SNV	exon26:c.A3142G:p.I1048V
**P_101**	VUS	H	48802333	-	-	-	exonic	missense SNV	exon14:c.G1622A:p.C541Y
**P_113**	VUS	H	48936954	-	-	0.00001627	exonic	missense SNV	exon2:c.C13T:p.R5C
**P_113**	Pathogenic	H	48704912	-	-	-	exonic	stopgain	exon65:c.C8080T:p.R2694X
**P_123**	VUS	H	48936888	-	-	-	exonic	missense SNV	exon2:c.G79A:p.A27T
**P_123**	VUS	H	48802247	-	-	-	exonic	missense SNV	exon14:c.T1708C:p.C570R
**P_136**	VUS	H	48808487	-	-	-	exonic	missense SNV	exon11:c.G1220A:p.G407D
**P_136**	Pathogenic	H	48704912	-	-	-	exonic	stopgain	exon65:c.C8080T:p.R2694X

Priority: H: high; cytoband: The chromosome segment where the mutation sites are located (observed by Giemas staining); POS:Absolute position of the mutation site on chromosome; Genomic, Esp6500siv2 and gnomAD: The frequency of newly discovered sites in the gnomAD, Genomic, and Esp6500siv2 databases; Func: Note the region of the mutation site; ExonicFunc: SNV or InDel variation types of exons; AAChange: amino acid change. The mutations in HGMD were shown in Red while other mutations shown in Black.

**Table 3:** Data filtering results of double mutation sites in eight MFS patients

Patients	Whether_freq_ 0.001	Whether_freq_ 0.0001	Genomic	Esp6500siv2	GnomAD	ACMG_classfication	Priority
**P_7**	TRUE	FALSE				VUS	H
**P_7**	TRUE	TRUE				LikelyPathogenic	H
**P_33**	TRUE	FALSE				VUS	H
**P_33**	TRUE	TRUE				Pathogenic	H
**P_41**	TRUE	TRUE				Pathogenic	H
**P_41**	FALSE	FALSE				VUS	H
**P_76**	TRUE	FALSE				VUS	H
**P_76**	TRUE	FALSE				VUS	H
**P_101**	TRUE	TRUE				VUS	H
**P_101**	TRUE	TRUE				VUS	H
**P_113**	TRUE	TRUE				Pathogenic	H
**P_113**	TRUE	FALSE				VUS	H
**P_123**	TRUE	TRUE				VUS	H
**P_123**	FALSE	FALSE				VUS	H
**P_136**	TRUE	TRUE				Pathogenic	H
**P_136**	TRUE	TRUE				VUS	H

Whether_freq_0.001: Whether the locus is less than 0.001 or has no record in gnomAD data (gnomAD_ALL and gnomAD_EAS) and in house Novo-Zhonghua exome database from Novogene; Whether_freq_0.0001:Whether the locus is less than 0.0001 or has no record in gnomAD data (gnomAD_ALL and gnomAD_EAS) and in house Novo-Zhonghua exome database from Novogene. True means yes and False means no. Genomic, Esp6500siv2 and gnomAD**:** The frequency of newly discovered sites in the gnomAD, Genomic, and Esp6500siv2 databases;

### Two-mutations in *FBN1* gene

We found eight patients (5 males and 3 females) had more than one SNP or INDEL on *FBN1* (Table 2). The age of the patients with MFS related symptoms was under 35 years old. Only P_123 didn’t present cardiovascular complications, but the patient had high myopia and dislocated lens. In order to further ensure the accuracy of two mutations sites, we set more strict filter conditions (set the data filtering standard of 0.001 / 0.0001, as shown in Table 3), and finally determined that only P_101 (exon26:c.A3142G:p.I1048V/exon14:c.G1622A:p.C541Y) and P_136 (exon11:c.G1220A:p.G407D/exon65:c.C8080T:p.R2694X) have two mutations in the *FBN1* gene at the same time (According to the incidence rate of 1/10000 in MFS patients, two mutations were confirmed). Both patients presented with widened aorta, and the younger brother of P_136 was also an MFS patient, but the phenotype was not as obvious as in patient P_136. MFS onset in the two patients was at a relatively earlier age, P_101 disease onset at 6 years of age and P_136 at 7 years.

### The second gene mutation

Surprisingly the sequencing results revealed the presence of mutations in genes other than the *FBN1* gene. These genes were *PKD1, PKD2, FLNA, NKX2-5* and *ACVRL1*. Mutations in *PKD1* gene appeared at much higher frequency than others. Twenty-seven patients (13 males and 14 females) have mutations in *PKD1* which is located on chromosome 16 in humans. The average age of disease onset is 27.27 years in these patients. Sixteen of the patients suffered from cardiovascular complications. Additionally we found that 14 of 27 patients have missense mutations in *FBN1*. Although mutations in other genes (*MFAP5, SMARTC2*, and*SMAD3*) have been detected but they didn't appear to contribute to MFS according to the low pathogenicity after filtering.

### Analysis of genotype–phenotype correlations in MFS

**Figure 1:**
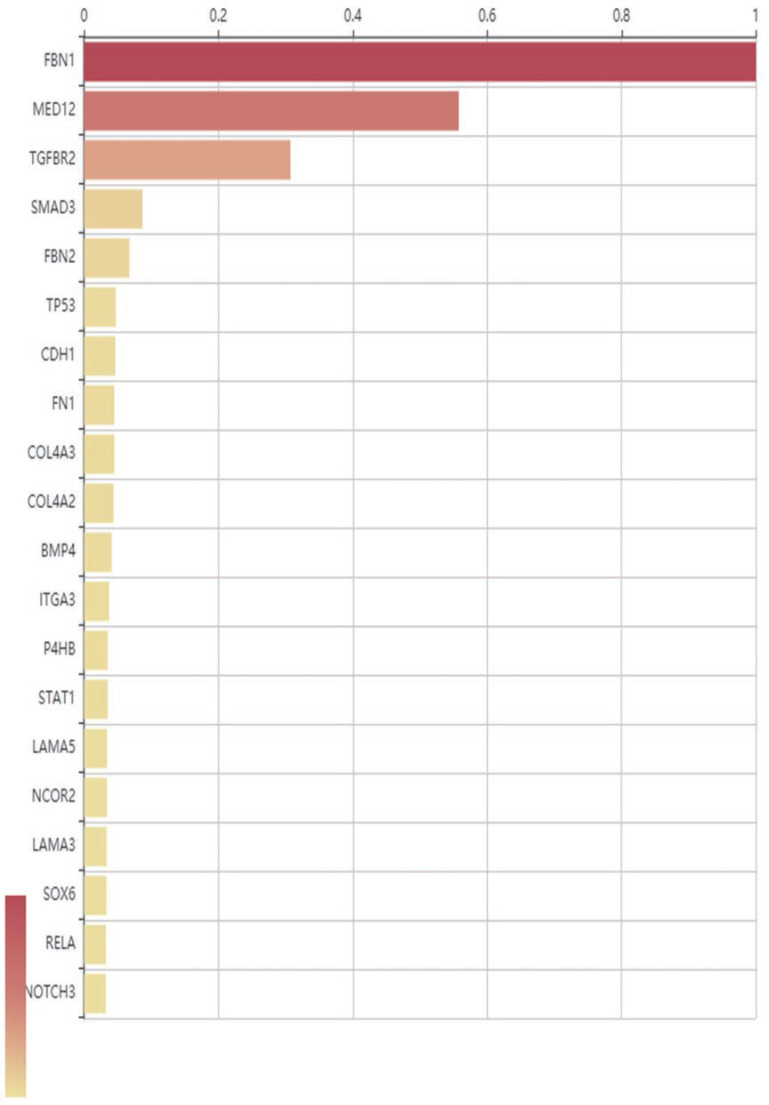
Correlations between mutated genes and MFS according to sequencing results Only the top 20 mutated genes were shown. The maximal relevance score is 1, and the ranking is based on the built-in rules.

In order to construct a genotype–phenotype correlations map, the sequencing results along with various databases were combined to screen and sort the affected genes. Genes were sorted based on the strength of the correlation between genetic mutations and MFS (Figure 1). The top 10 candidate genes associated with phenotype or disease are: *FBN1, MED12, TGFBR2, SMARD, FBN2, TP53, CDH1, FN1, COL4A3*, and COL4A2.

**Figure 2:**
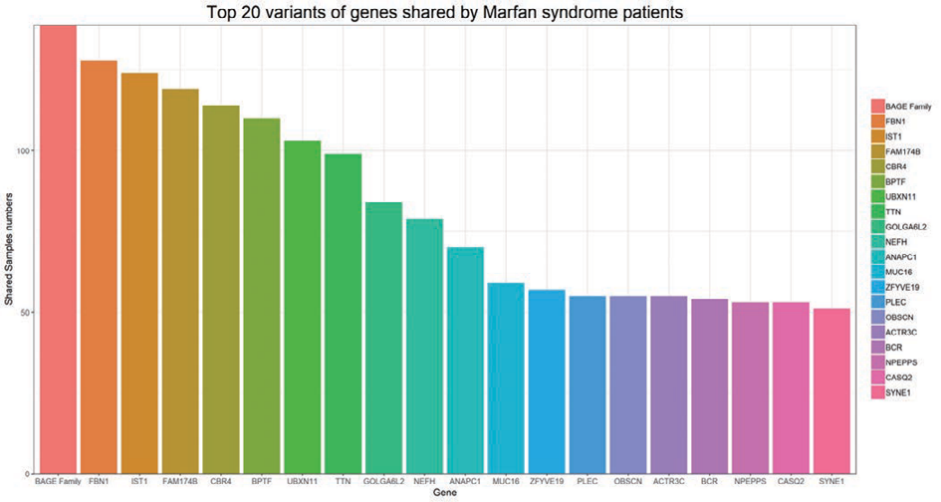
Top 20 variants of common genes mutated in patients with MFS The top 10 candidate genes associated with phenotype or disease are: FBN1, MED12, TGFBR2, SMARD, FBN2, TP53, CDH1, FN1, COL4A3, COL4A2. Tthere are many genes related to cardiomyopathy, such as TTN, NEFH, PLEC, CASQ2, SYNE1.

**Figure 3:**
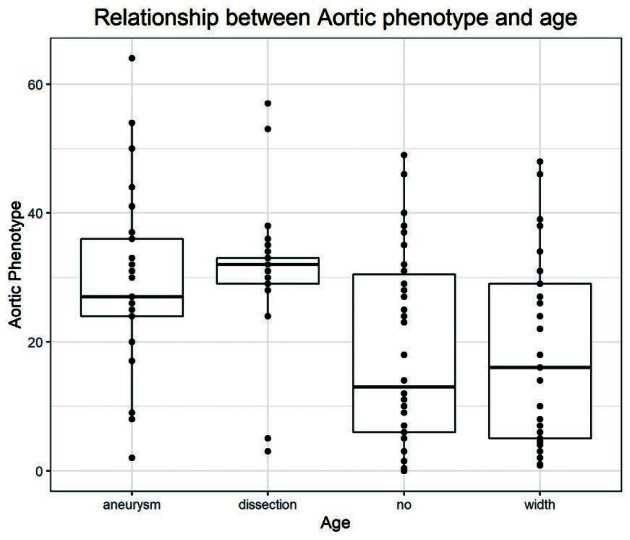
Correlations between aortic phenotype and age According to the patient’s aortic phenotype, the patients were divided into four groups. The correlation was mainly observed in patients between 20 and 40 years of age. Whereas the age distribution of the patients with no aortic phenotype or aortic width had a wider range with more patients younger than 20 years of age.

Data analysis also indicated that there are many genes related to cardiomyopathy, such as *TTN, NEFH, PLEC, CASQ2*, and *SYNE1* (Figure 2). At the same time patients with the aortic dissection and aneurysm were further studied based on the relationship between the age at disease onset and the occurrence of aortic events (divided into four aortic phenotypes of no, width, aneurysm, and dissection). The correlation was mainly observed in patients between 20 and 40 years of age. Whereas the age distribution of the patients with no aortic phenotype or aortic width had a wider range with more patients younger than 20 years of age (Figure 3).

Statistical analysis using *t* test revealed a significant difference between the age of patients with no aortic phenotype and those with aortic aneurysm and aortic dissection (*P*<0.05). Whereas no significant difference was found between the age of patients showing no aortic phenotype and those with only aortic widening (*P*>0.05) (Table 4).

**Table 4:** Age comparison of patients in different aortic phenotype groups

*P* value	No aortic phenotype
**Width**	0.6075
**Dissection**	0.004868
**Aneurysm**	0.000214

Width: aortic widening; Aneurysm: aneurysm of aorta; Dissection: aortic dissection.

The patients harboring two-mutations in the*FBN1* gene showed prominent MFS phenotypes compared to other family members. Moreover, the external manifestations of patients are also obvious, but not every patient has aortic complications.

## Discussion

Pathogenic mutations in the *FBN1* gene have been reported to cause MFS pathologies affecting peripheral tissues, skeletal anomalies and aortic complications [1]. Among the 131 Marfan patients enrolled in this study, 38 patients were found in HGMD database to harbor novel mutations in *FBN1* loci. The findings in the present study expand the spectrum of the *FBN1* site mutation information database. The novel loci were not detected in the early stage of genomic testing [5]. It is proved that WES technology is superior to panelgenomic testing for the discovery of new mutation sites in MFS patients, and the coverage is more extensive, but due to the current testing costs, WES technology is subject to certain restrictions [5]. However, with the advancement of science and technology, the current testing costs have dropped to affordable levels. The present study provides additional evidence that WES technology should be widely used in clinical and scientific research for more accuracy and sensitivity.

To this extent, one of our novel findings is the detection of two mutations in the *FBN1* gene that exacerbate the disease pathology in MFS patients. The mutations we report in the present study are: exon26:c.A3142G:p.I1048V and exon14:c.G1622A:p.C541Y from one patient (P_101), exon11:c.G1220A:p.G407D and exon65:c.C8080T:p.R2694X from a second patient (P_136). Previous reports indicate that all patients with two mutations in *FBN1* have obvious MFS phenotypes. Nonetheless the incidence is extremely low in the population, and the clinical significanceis still unknown [23,24]. In our study, patients with two mutations in *FBN1* manifested external phenotypes and early disease onset, which is consistent with previous reports [6]. Although the specific mechanism through which the two mutations occur in *FBN1* in MFS patients is still not clear, the current reports pointed out that the probability for such mutation to cause severe MFS is very high [24,25,26]. This highlights the importance of evaluating patients with severe MFS, early onset and high penetrance for the presence of two mutations in the *FBN1* gene.

In this group of MFS patients, we also found 27 patients with *PKD1* gene mutations, but the mutations are located on the patient’s chromosome 16. Previously, it has been reported that *PKD1* mutation is one of the main genes that cause polycystic kidney disease in patients. The pathogenic genes are all located on chromosome 15 of patients, and the disease is a late-onset disorder [27,28]. In recent years, it has been found that some patients with *PKD1* gene mutation suffers from aortic dissection [29]. Although *PKD1* gene mutation and aortic related complications were found in some patients, polycystic kidney did not appear in these patients. In addition, *PKD1* gene mutations in this group of patients are located on chromosome 16, so far, there is no report on whether the mutation of *PKD1* gene in addition to chromosome 15 will also cause polycystic kidney disease in MFS patients. Moreover, polycystic kidney disease is a late onset disease while MFS patients are all early onset, in which the appearance of other phenotypes still need further follow-up, but we did not find any renal lesions in the existing older MFS patients. Therefore, the detection of this genetic mutation in patients with MFS, whether it alone causes aortic related complications, or whether it works together with *FBN1* gene, needs further research and demonstration.

Although peripheral tissues of Marfan patients are often affected, aortic complications are the main mortality risk factors. Previous studies have shown that MFS patients with severe phenotypes are prone to mutations in the *FBN1* gene in the exon 24-32 region [7]. Early research in our center also showed that patients with *FBN1* frameshift mutations and nonsense mutations are prone to aortic dissection, and missense mutations are prone to aortic aneurysms [8]. Based on the observation that older patients in the same family seemed to have either aortic aneurysm or aortic dissection wefurther analyzed data considering age as a variable. Patients were grouped in four categories based on the aortic phenotype. Group-1: aortic widening; Group-2: aortic aneurysm; Group-3: aortic dissection and Group-4: No aortic phenotype (no). The correlation between the aortic phenotype and age was significant and obvious as seen in the box plot (Figure 3). This study found that patients with aortic aneurysms and dissections are between 20 and 40 years of age. This finding highlights the importance of being more vigilant for MFS patients who are in this age group to prevent the occurrence of aortic adverse events.

In the present study, only exons from MFS patient’s DNA were studied, but related reports showed that MFS can also be caused by mutations in the intron region of the *FBN1* gene, and these mutations can have an impact on the prognosis and phenotype of patients. We are working to obtain a large sample size and multicenter data to analyze gene information of introns in patients with MFS in the near future in order to improve genetic information of patients with MFS. And, for other gene loci in the article, such as PKD1, etc., since there are no samples during data analysis, we cannot perform sanger verification for these genes. However, as shown by the data provided, these sites’ sequencing data are good (such as total depth and mutation depth etc.). We believe that these site mutations are very reliable. However, we will study this part in-depth in the future.

## Conclusion

In the present study, through the whole-exome sequencing of MFS patients, we found many new MFS mutation sites and patient double mutation sites, which further verified the pathogenicity of the FBN1 gene to MFS patients. At the same time, it is believed that FBN1 gene mutation, which the main factor leading to patients with aortic dissection or aneurysm, and double mutation sites are a crucial factor that aggravates the patient’s phenotype. Besides, we also found that the age of MFS patients with related aortic complications in the same family is generally between 20 and 40 years, which is older than other MFS patients in the same family with no or mild aortic phenotype, which can provide a basis for clinicians to have better disease prognosis and prevent the occurrence of aortic adverse events. The clinical difference of MFS patients is vast, which may be caused by modification factors, such as the gene loci found on PKD1 in this sequencing. We consider these loci may be modified factors, but they do not occur on the disease-causing chromosomes. Due to the lack of samples in this part of the sequencing process, we cannot verify this part of the data. However, for this research, we are only preliminary research, and the relationship between these factors needs further research and discussion in the future.

## Data Availability

Data are available upon request.

## Abbreviations

ACMG: American College of Medical Genetics and Genomics
*FBN1*: fibrillin 1
MFS: Marfan syndrome
VUS: variant of uncertain significance
